# Dynamical alterations of brain function and gut microbiome in weight loss

**DOI:** 10.3389/fcimb.2023.1269548

**Published:** 2023-12-20

**Authors:** Jing Zhou, Xiaoling Wu, Tianyuan Xiang, Fei Liu, Hui Gao, Li Tong, Bin Yan, Zhonglin Li, Chi Zhang, Linyuan Wang, Lei Ou, Zhongxia Li, Wen Wang, Tingting Yang, Fengyun Li, Huimin Ma, Xiaojuan Zhao, Na Mi, Ziya Yu, Canhui Lan, Qi Wang, Hao Li, Liming Wang, Xiaoning Wang, Yongli Li, Qiang Zeng

**Affiliations:** ^1^ Henan Provincial Research Center of Clinical Medicine of Nephropathy, Henan Provincial People’s Hospital, Zhengzhou University People’s Hospital, Henan University People’s Hospital, Zhengzhou, China; ^2^ Department of Nuclear Medicine, Henan Key Laboratory of Chronic Disease Health Management, Henan Provincial People’s Hospital, Zhengzhou University People’s Hospital, Zhengzhou, Henan, China; ^3^ Health Management Institute, The Second Medical Center & National Clinical Research Center for Geriatric Diseases, Chinese People's Liberation Army (PLA) General Hospital, Beijing, China; ^4^ Institute of Microbiology, Chinese Academy of Sciences, Beijing, China; ^5^ Henan Key Laboratory of Imaging and Intelligent Processing, People’s Liberation Army (PLA) Strategic Support Force Information Engineering University, Zhengzhou, Henan, China; ^6^ Department of Radiology, Henan Provincial People’s Hospital, Zhengzhou, Henan, China; ^7^ BYHEALTH Institute of Nutrition & Health, BYHEALTH Co. Ltd, Guangzhou, Guangdong, China; ^8^ Department of Cardiology, Sun Yat-sen Memorial Hospital of Sun Yat-sen University, Guangzhou, Guangdong, China; ^9^ Department of Nutrition, Henan Provincial People’s Hospital, Zhengzhou University People’s Hospital, Henan, Zhengzhou, China; ^10^ Department of Health Management, Henan Provincial People’s Hospital, Zhengzhou University People’s Hospital, Henan University People’s Hospital, Zhengzhou, Henan, China; ^11^ Beijing Rexinchang Biotechnology Research Institute Co. Ltd, Beijing, China; ^12^ Cuiying Biomedical Research Center, Lanzhou University Second Hospital, Lanzhou, Gansu, China; ^13^ Department of Health Management, Fuwai Central China Cardiovascular Hospital, Zhengzhou, China; ^14^ The Institute of Geriatrics, The State Clinic Center for Geriatrics & The State Key Laboratory of Kidney, The People’s Liberation Army (PLA) General Hospital, Beijing, China; ^15^ Department of Health Management, Henan Key Laboratory of Chronic Disease Management, Henan Provincial People’s Hospital, Zhengzhou University People’s Hospital, Henan University People’s Hospital, Zhengzhou, Henan, China

**Keywords:** brain-gut-microbiome axis, weight loss, functional magnetic resonance imaging, intermittent energy restriction, metagenomics

## Abstract

**Objective:**

Intermittent energy restriction (IER) is an effective weight loss strategy. However, little is known about the dynamic effects of IER on the brain-gut-microbiome axis.

**Methods:**

In this study, a total of 25 obese individuals successfully lost weight after a 2-month IER intervention. FMRI was used to determine the activity of brain regions. Metagenomic sequencing was performed to identify differentially abundant gut microbes and pathways in from fecal samples.

**Results:**

Our results showed that IER longitudinally reduced the activity of obese-related brain regions at different timepoints, including the inferior frontal orbital gyrus in the cognitive control circuit, the putamen in the emotion and learning circuit, and the anterior cingulate cortex in the sensory circuit. IER longitudinally reduced *E. coli* abundance across multiple timepoints while elevating the abundance of obesity-related *Faecalibacterium prausnitzii, Parabacteroides distasonis*, and *Bacterokles uniformis*. Correlation analysis revealed longitudinally correlations between gut bacteria abundance alterations and brain activity changes.

**Conclusions:**

There was dynamical alteration of BGM axis (the communication of *E. coli* with specific brain regions) during the weight loss under the IER.

## Introduction

Obesity is a challenging public health issue worldwide (Schwartz et al., 2017). Higher-order executive functions play important roles in successful weight loss and weight maintenance (Torres–Fuentes et al., 2017; van de Wouw et al., 2017; Frank et al., 2021). Functional magnetic resonance imaging (fMRI) studies have revealed that addiction-related brain regions, including the reward, cognitive control, emotional, and sensory circuits, contributes to the pathogenesis of obesity and weight management by regulating eating behavior. The reward circuit including striatum, nucleus accumbens and ventral tegmental area, also known as appetite network, is mainly responsible for regulating the motivation for food (Torres–Fuentes et al., 2017; van de Wouw et al., 2017; Frank et al., 2021). The cognitive control circuit including dorsolateral prefrontal cortex (DLPFC) and anterior cingulate gyrus (ACC), is responsible of self-limiting calorie intake (Torres–Fuentes et al., 2017; van de Wouw et al., 2017; Frank et al., 2021). The emotional circuit is responsible for the affective learning and memory of food cue (Torres–Fuentes et al., 2017; van de Wouw et al., 2017; Frank et al., 2021). The main nuclei include amygdala, hippocampus and putamen (Torres–Fuentes et al., 2017; van de Wouw et al., 2017; Frank et al., 2021). The sensory circuit including orbito frontal cortex (OFC) and insula drive food intake and consumption (Torres–Fuentes et al., 2017; van de Wouw et al., 2017; Frank et al., 2021). For example, Hermann et al. have found that the striatum in the reward circuit responds more strongly to high-calorie food at the early stage of calorie restriction, predicting weight trajectory and weight-loss intervention outcomes (Hermann et al., 2019). Neseliler et al. have shown that the activity of the dorsolateral prefrontal cortex in cognitive control circuit increases at one month after calorie restriction and returned to baseline at three months after calorie restriction, positively correlating with weight loss (Neseliler et al., 2019). However, the mechanism underlying the chronological changes of the brain activity during calorie restriction remains unclear.

The central nervous system interacts with gut microbes through a bidirectional brain-gut-microbiome (BGM) axis. Food intake affects the activity of brain circuits that control energy balance (Clemmensen et al., 2017). Abnormal response of the brain circuits to nutritional signals alters eating behavior, contributing to the development of obesity (Martin et al., 2018). Gut microbes regulate brain activity and eating behavior through multiple mechanisms. Neurotransmitters and neurotoxins produced by gut microbes in pathological states have access to the central nervous system through the blood-brain barrier, affecting brain function and then eating behavior (Holder and Chassaing, 2018). Metabolic disorders resulting from the alteration in gut microbiota composition may affect the activity of the hypothalamus and other diet-related brain regions through the vagus nerves or systemic circulation (Mishra et al., 2016; Mohajeri et al., 2018). Therefore, in obesity management, the alteration in gut microbiota composition resulting from dietary intervention may maintain energy homeostasis by restoring BGM communication, thereby improving weight loss (van Son et al., 2021). However, there is still a lack of understanding about the dynamic changes and the underlying pathways of the BGM axis in dietary intervention.

Intermittent energy restriction (IER) is defined as periods of restricted energy intake followed by periods of normal energy intake. Studies have shown that long-term IER (> 3 months) is an effective weight loss strategy (Harvie and Howell, 2016; Schübel et al., 2018; Dong et al., 2020). In this study, using fMRI and metagenomic sequencing, we investigated the dynamic effects of a short-term (2-month) IER intervention on the BGM axis in obese individuals. Our study identified differentially abundant gut microbes and potential pathways underlying the temporal changes in the BGM axis in response to IER, providing new information about the role of the BGM axis in weight loss.

## Methods

### Study approval

This study was approved by the Ethics. This study was registered at https://clinicaltrials.gov. All procedures were performed following relevant ethical regulations, Helsinki Declaration, and privacy legislation. All participants provided written informed consent.

### Subjects

A total of 25 obese adults with body mass index (BMI) ranging from 28 to 45 kg/m^2^ were recruited from hospitals from April to November 2018. The recruitment process has shown in **Supplementary Figure S1**. All patients had well-controlled hypertension, diabetes, hyperlipidemia, hypeluricemia, or metabolic syndrome. The individuals with serious cardiopathy, liver dysfunction, renal disease, hypoglycemia, anemia, malnutrition, hemopoietic system disease, systemic immune system disease, infectious disease, or neurological disorders were excluded. All participants provided written informed consent.

### IER protocol

**Figure 1:**
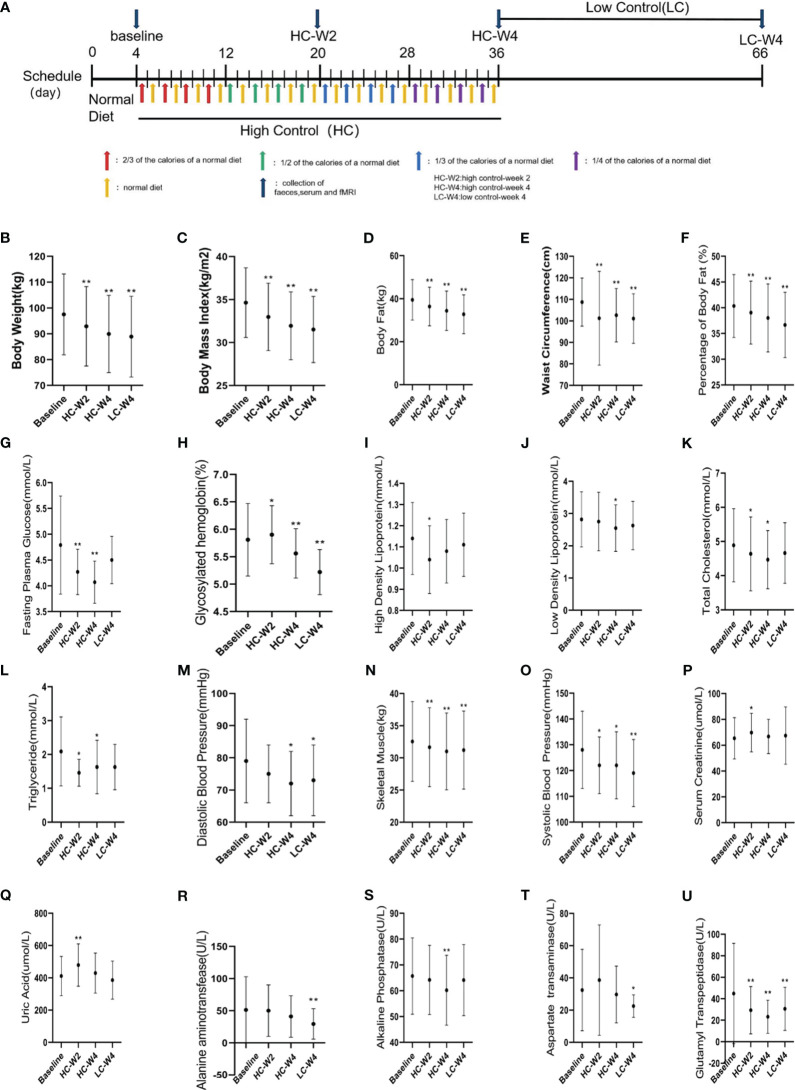
Clinical parameters of participants. **(A)** Study design. A total of 35 obese adults completed a three-phase intermittent energy restriction (IER). In the 4-day phase I, the subjects were on normal diet without restriction on calories or food types. In the 31-day highly-controlled phase II, the participants were on a diet at 2/3, 1/2, 1/3, and 1/4 of the normal caloric intake every other day for 7–8 days, respectively. In the 30-day lowly-controlled phase III, the participants were on a calorie-restricted diet (600 kal/day for men, and 500 kal/day for women) every other day. A total of 25 participants with obesity successfully achieved weight loss after intermittent energy restriction (IER) intervention. **(B–U)** Obesity-related clinical parameters were measured at baseline, midpoint and endpoint of phase II, and endpoint of phase III. HC-W2, midpoint of highly-controlled phase II; HC-W4, endpoint of highly-controlled phase II; LC-W4, endpoint of lowly-controlled phase III. Data are expressed as the mean ± standard deviation. *P < 0.05, **P < 0.01 *vs*. baseline; n = 25.

As shown in **Figure 1A**, in phase I, the participants were on normal diet without restriction on calories and food types for four days. The average daily energy intake of each subject was recorded according to 24-h dietary recalls for four days as their basic energy intake (Park et al., 2018). The IER meals were formulated by a clinical dietitian based on each participant’s basic energy intake and provided to each participant on the energy-restricted diet day. Each meal consisted of 55% carbohydrates, 15% protein and 30% fat. The phase II which is the high-controlled fasting phase included 32 days (8 d each stage, 4 stages in total).At stage 1, 2, 3 and 4, patients were provided with 2/3, 1/2, 1/3 and 1/4 of each participant’s basic energy intake respectively. Patients ate independently without restriction on every other unrestricted energy intake day at home. The Phase III was the low-controlled fasting phase which includes 30 days. During the Phase III, the meals were not provided to the participants. They were gave a list of food (consisted of 55% carbohydrates, 15% protein and 30% fat) to have and asked to have energy-restricted diet (600 call/day for men, 500 call/day for women) on the alternating day at home. The feces and blood samples were collected before phase I (baseline), at the midpoint (HC-W2) and endpoint (HC-W4) of phase II, and at the endpoint of phase III (LC-W4).

### Measurement of clinical parameters

Body weight, waist circumference, body fat, systolic blood pressure, and diastolic blood pressure were measured at baseline, midpoint and endpoint of phase II, and endpoint of phase III. Blood samples were collected in the morning after overnight fasting at different timepoints as above mentioned. Serum was obtained by centrifugation at 3,000 rpm for 15 min at 23°C and stored at -80°C until use. Serum levels of fasting plasma glucose, glycosylated hemoglobin, total cholesterol, glutamyl transpeptidase, high-density lipoproteins, low-density lipoproteins, aspartate aminotransferase, alanine aminotransferase, alkaline phosphatase, creatinine, and uric acid were measured by the Laboratory of Hospital.

### fMRI

Brain activity was recorded by resting-state fMRI. The parameters were Repeat time (TR) = 2000 ms, Echo Time (TE) = 35 ms, Field of View (FOV) = 220 mm×220 mm, matrix size = 94×94, slices = 75, slice thickness = 2.2 mm, flip angle = 80°, and Simultaneous MultiSlice (SMS) factor = 3. The regional homogeneity (ReHo) was measured based on Kendall’s coefficient concordance as previously described (Zang et al., 2004). Data were analyzed using DPABI (http://rfmri.org/dpabi) and SPM12 (www.fil.ion.ucl.ac.uk/spm). Briefly, DICOM-format data were converted to NIFTI-format. After discarding the 10 initial scans, data were subjected to slice-timing and head motion correction. Data were then normalized to the Montreal Neurological Institute standard space by linearly registering using echo-planar imaging (EPI) template. After smoothing the data using a 6 mm full width at half maximum, data were detrended to eliminate the linear trend of time courses and filtered with low frequency fluctuations (0.01-0.08 Hz). Six head motion parameters, white matter mask, the whole-brain mask, and cerebrospinal fluid mask were regressed out of the EPI time series. Five participants with abnormal head motion parameters (displacement > 2 mm and/or rotation > 2 degrees) were excluded.

The resting-state ReHo maps were collected. The ReHo values were compared using paired t-test. The region of interest was selected as follows: first, the peak point of the paired t-test results was set as the center of the region of interest; second, the brain regions that were not included in the paired t-test but important for obesity research were selected. The regions of interest were stored as the mask of seed points by Anatomical Automatic Labeling (AAL) template, followed by loading into the results of the paired t-test. The activity of corresponding brain regions was obtained, and the peak coordinates and t values of the brain regions were found.

### Metagenomic sequencing

Fecal samples from 25 participants who successfully lost weight were subjected to metagenomic sequencing. Genomic DNA was isolated from fecal samples using Magen Magpure stool DNA KF kit B (Magen, China) following the manufacturer’s instructions. A paired-end DNA library with insert size of 250 bp was constructed. The DNA libraries were sequenced with paired-end reads of 2 × 100 bp on the BGISEQ-500 platform (BGISEQ, China).

Sequencing data were processed using KneadData v0.7.2 (https://bitbucket.org/biobakery/kneaddata/wiki/Home) as previously described (Liu et al., 2021). Briefly, the raw reads with 50% low quality bases (quality ≤ 20) or more than five ambiguous bases were excluded. The remaining reads were mapped to the human genome (hg19) using Short Oligonucleotide Analysis Package (SOAP) v2.22 (-m 100 -x 600 -v 7 -p 6 -l 30 -r 1 -M 4 -c 0.95), and the matching reads were removed. The high-quality non-human reads were considered clean reads. The clean reads were aligned against 11.4 M human gut microbial gene catalog using SOAP v2.22 (-m 100 -x 600 -v 7 -p 6 -l 30 -r 1 -M 4 -c 0.9) to generate the gene abundance profile. To obtain the taxonomic profiles, metaphlan2 (–input_type fastq –ignore_viruses –nproc 6) was used to generate species profile.

The raw sequence data reported in this paper have been deposited in the Genome Sequence Archive (Genomics, Proteomics & Bioinformatics 2021) in National Genomics Data Center (Nucleic Acids Res 2022), China National Center for Bioinformation / Beijing Institute of Genomics, Chinese Academy of Sciences (GSA: CRA013299) that are publicly accessible at https://ngdc.cncb.ac.cn/gsa (Chen et al., 2021; CNCB-NGDC Members and Partners, 2023).

### Gut microbiota diversity analysis

Alpha diversity (within samples) was assessed by Shannon index. Beta diversity (between samples) was assessed by Bray-Curti’s dissimilarity based non-metric multidimensional scaling (NMDS). Data were analyzed using R 3.2.5, vegan package 2.4-4.

### Differentially abundant microbial species and pathways

Differentially abundant Gut microbial species and pathways were identified by linear discriminant analysis (LDA). Logarithmic LDA scores threshold was 2.0. Kruskal-wallis test threshold was *P* < 0.05.

### Statistical analysis

The measurement data were expressed as the mean ± standard deviation. Statistical analysis was performed using SPSS version 17.0 (SPSS, Chicago, IL, USA). Data with normal distributions were analyzed using ANOVA and two-tailed Student’s t-test. Nonnormal distributions were compared using Mann-Whitney, Kolmogorov-Smirnov, or Wald- Wolfowitz test. The ReHo values were compared using paired t-test and subjected to Gaussian random field correction. NMDS data was compared using Wilcoxon rank-sum test and Kruskal-wallis rank sum test. *P* < 0.05 was considered statistically significant. The correlations between gut microbiota and fMRI results were analyzed by Spearman’s rank correlation. Spearman`s correlation coefficient ≥ 0.2 and *P* < 0.05 were considered significant correlation.

## Results

### IER reduces obesity and improves obesity-related clinical parameters

As shown in **Supplementary Figure S1**, a total of 41 participants were recruited, 35 completed the IER, and 25 successfully lost weight as evidenced by continuous, significant reductions in body weights during IER intervention (all *P* < 0.01 *vs*. baseline; **Figure 1B**). In the 25 participants, by comparing with baseline parameters, we observed sustained, significant reductions in body mass index, body fat, waist circumference, percent of body fat, skeletal muscle (all *P* < 0.001), systolic blood pressure (all *P* < 0.05), and serum levels of glycosylated hemoglobin (all *P* < 0.05) and glutamyl transpeptidase (all *P* < 0.01) during IER intervention. The body weight of obese participants after IER intervention were significantly lower than those before intervention[(89.92 ± 14.98) *vs.* (97.53 ± 15.67) kg, *P* < 0.001]. In addition, diastolic blood pressure and serum levels of fasting plasma glucose, total cholesterol, high-density lipoproteins, low-density lipoproteins, alanine aminotransferase, aspartate aminotransferase, and alkaline phosphatase were significantly decreased on at least one time point during IER (all *P* < 0.05; **Figures 1C–U**). These data suggest that IER not only reduces obesity but also alleviates obesity-related comorbidities such as hypertension, hyperlipidemia, and liver dysfunction.

### IER intervention reduces the activity of brain regions related to eating behavior

**Figure 2:**
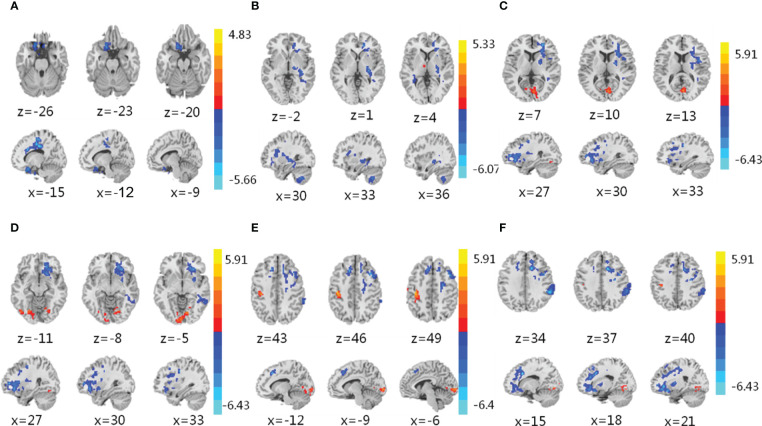
Functional magnetic resonance imaging (fMRI). **(A–F)** fMRI was conducted to assess brain activity in the left inferior frontal orbital gyrus **(A)**, putamen **(B)**, right putamen **(C)**, right inferior frontal orbital gyrus **(D)**, left dorsolateral prefrontal cortex **(E)**, and anterior cingulate cortex **(F)** of obese participants in response to IER. The regional homogeneity (ReHo) was measured to compare brain activity at midpoint of phase II **(A)**, upper), endpoint of phase II **(B)**, upper), and endpoint of phase III **(C–F)**, upper) with that at baseline **(A–F)**, lower). Blue denotes decreased ReHo values. Red denotes increased ReHo values. z: the number of axial layers; x: the number of sagittal layers.

Then, we performed resting-state fMRI to explore the effect of IER on brain activity. Representative fMRI images are shown in **Figures 2A–F**. Statistical analysis of ReHo values is shown in **Table S1**. We found significant decreases in the Reho values of the left inferior frontal orbital gyrus (**Figure S2D**) and putamen (**Figure S2B**) at midpoint and endpoint of phase II, respectively, compared with those at baseline. We also observed significant decreases in the Reho values of the right inferior frontal orbital gyrus (**Figure S2A**), anterior cingulate cortex (**Figure S2C**), left dorsolateral prefrontal cortex (**Figure S2D**), and right putamen (**Figure S2F**) at endpoint of phase III, compared with those at baseline (**Figure S2**). These brain regions are responsible for cognitive control, emotion and learning memory, as well as sensory (Volkow et al., 2003; Dagher, 2012; Ochner et al., 2013; Gettens and Gorin, 2017). No significant changes were observed in brain activity of the reward circuit or other brain regions (**Figure S2**). These data suggest that IER reduces the activity of brain regions related to the regulation of food intake, thus contributing to successful weight loss and maintenance.

### IER increases gut microbial diversity

**Figure 3:**
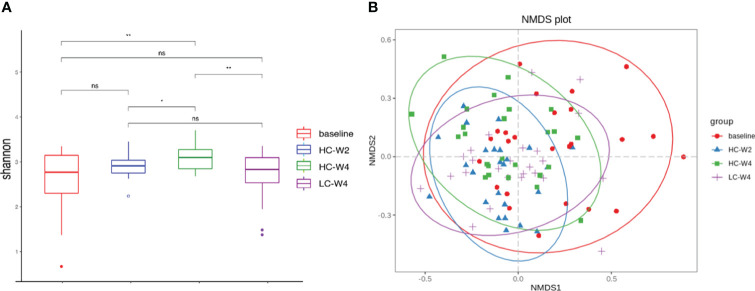
Diversity analysis of gut microbiota based on metagenomic sequencing. **(A)** Shannon indexes (alpha diversity) of the gut microbiota at the species level. *P < 0.05, **P < 0.01; n = 25. **(B)** Bray-Curtis dissimilarity (beta diversity) based on non-metric multidimensional scaling. NMDS, non-metric multidimensional scaling. ns, none significance.

To explore the effect of IER on gut microbiota in obese patients, we performed metagenomic sequencing and bioinformatics analyses. Shannon diversity analysis at the species level showed that the gut microbial species at endpoint of phase II was significantly increased compared with that at baseline or midpoint of phase II. The gut microbial species at the end of phase III was significantly decreased compared with that at the end of phase II. No significant difference was observed in the gut microbial diversities between baseline and the end of phase III (**Figure 3A**). Bray-Curtis dissimilarity analysis showed that the gut microbiota of obese individuals at phase II and phase III clustered together (**Figures 3B**, **S3B**). These data suggest that IER intervention increases gut microbial richness and diversity in obese individuals.

### IER dynamically alters the abundance of obesity-related gut bacteria

**Figure 4:**
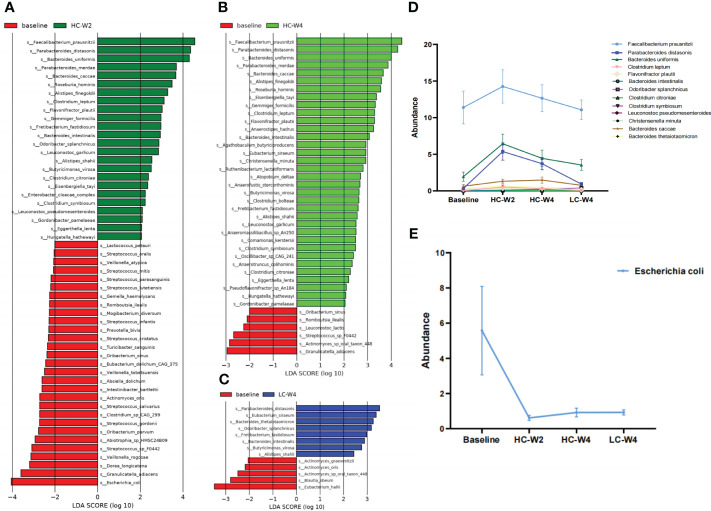
Identification of differentially abundant gut bacteria during IER. **(A–C)** Linear discriminant analysis (LDA) was performed to identify differentially abundant gut bacteria at midpoint of phase II **(A)**, endpoint of phase II **(B)**, and endpoint of phase III **(C)** compared with those at baseline. The species with LDA score (log 10) > 2.0 and *P* < 0.05 (Kruskal-wallis test) were considered differentially abundant bacteria. **(D)** The abundance of representative differentially abundant gut bacteria during IER. **(E)** Gut *Escherichia coli* abundance during IER. LDA, linear discriminant analysis.

Next, we sought to identify differentially abundant gut microbial species and pathways during IER. The results of LDA showed that food-borne pathogenic *Escherichia coli* was the most abundant species in the gut microbiota of obese individuals at baseline (**Figures 4A–C**). IER dramatically reduced *E. coli* abundance across multiple timepoints (**Figure 4E**). On the other hand, compared with those at baseline, obesity-related *Faecalibacterium prausnitzii, Parabacteroides distasonis*, and *Bacterokles uniformis* were remarkably increased in gut microbiota of obese individuals during phase II (all *P* < 0.05; **Figure 4D**), peaking at midpoint of phase II and then gradually decreasing to near baseline levels. No significant differences were observed in the abundance of *F. prausnitzii* and *P. distasonis* between endpoints of phase II and phases III. Similar trends were observed in the abundance of *Clostridium leptum, Flavonifractor plautii, Bacterokles intestinalls, Odoribacter splanchnicus*, and *Clostridium citroniae* (**Figure 4D**).

### Gut microbiota alterations are associated with brain activity changes across different timepoints during IER intervention

**Figure 5:**
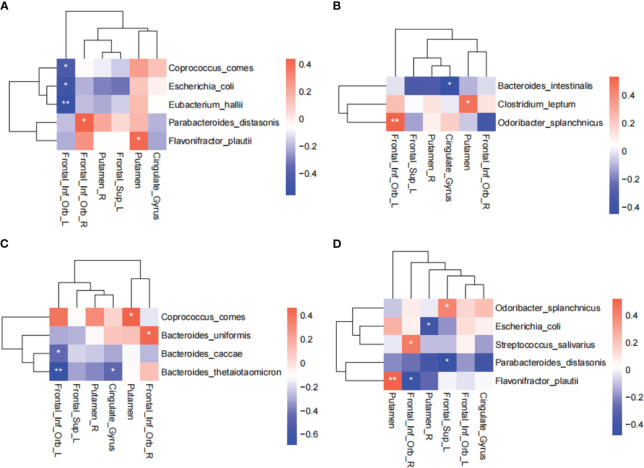
Correlation between differentially abundant gut bacteria and brain regions responding to IER. Spearman rank correlation coefficient was used to assess the correlation between differentially abundant gut bacteria and brain regions responding to IER at baseline **(A)**, midpoint **(B)** and endpoint **(C)** of phase II, and endpoint of phase III **(D)**. *P < 0.05, **P < 0.01. AAL Template names were used in all brain regions.

Considering the important role of the BGM axis in weight maintenance, we investigated the dynamic correlation between differentially abundant gut bacteria and the brain regions responding to IER. The results showed that at baseline, the abundance of *E. coli, C. comes*, and *E. hallii* were negatively correlated with the activity of left orbital inferior frontal gyrus whereas the abundance of *P. distasonis* and *F. plautii* were positively correlated with the activity of right orbital inferior frontal gyrus and right putamen, respectively (**Figure 5A**). At midpoint of phase II, the abundance of *B. intestinalis* was negatively correlated with the activity of anterior cingulate gyrus whereas the abundance of *C. leptum* and O*. splanchnicus* were positively correlated with the activity of putamen and left orbital inferior frontal gyrus, respectively (**Figure 5B**). At endpoint of phase II, the abundance of *B. caccae and B. thetaiotaomicron* were negatively correlated with the activity of left orbital inferior frontal gyrus, the *B. thetaiotaomicron* abundance was negatively correlated with the activity of anterior cingulate gyrus, and the *C. comes* abundance was positively correlated with the activity of putamen (**Figure 5C**). At endpoint of phase III, the abundance of *E. coli, P. distasonis, and F. plautii*were were negatively correlated with the activity of right putamen, left dorsolateral prefrontal lobe, and right orbital inferior frontal gyrus, respectively, whereas the abundance of *S. salivarius and F. plautii* were positively correlated with the activity of right orbital inferior frontal gyrus and putamen, respectively (**Figure 5D**). The differential microbes were *Coprococcus comes* at baseline, *Bacteroides uniformis* and *Parabacteroides distasonis* at HC-W2, and *Eubacterium Hallii* and *Agathobaculum butyriciproducens* at HC-W4 (**Figure S3**). These data suggest that IER induces a dynamic interaction between the brain and gut microbiota.

## Discussion

In this study, we described the dynamic effects of a short-term IER intervention on the BGM axis in obese individuals. We observed that IER induced constant, significant reductions in the activity of eating behavior-related brain regions, including the right inferior frontal orbital gyrus, anterior cingulate cortex, left dorsolateral prefrontal cortex, and right putamen. We also found that IER induced significant, dynamic changes in the abundance of some gut bacteria, including pathogenic *E. coli* and obesity-related probiotics *F. prausnitzii, P. distasonis*, and *B. uniformis*. Importantly, gut microbiota alterations correlated with brain activity changes across different timepoints in IER intervention. These data suggest that the dynamic interplay between the brain and gut microbiota plays an important role in weight loss.

We used fMRI to describe IER-induced dynamic changes in brain activity. At midpoint of phase II, we only observed significant reduction in the activity of the left inferior frontal orbital gyrus belonging to the OFC in the sensory and drive circuit. The OFC is related to the pleasure caused by the smell and taste of food, integrating sensory organs such as mouth, nose and eyes with fine-tune eating decisions through interaction with brain regions including thalamus, midbrain, and striatum (Seabrook and Borgland, 2020). Studies have shown enhanced OFC activity in obese persons in response to visual food cues compared with that in healthy controls (Pursey et al., 2014) and positive correlation between OFC activity and BMI (Batterink et al., 2010). In addition, OFC belongs to the emotion-driven “hot-system” that rapidly responds to environmental changes (Metcalfe and Mischel, 1999), consistent with our finding that OFC was the first brain region responding to IER. Thus, in our study, the decreased activity of the left inferior frontal orbital gyrus in the early phase of IER may promote weight loss by reducing food intake.

At endpoint of phase II, we observed a significant reduction in the activity of putamen in the emotion and learning memory network. Putamen is involved in learning and memory regards to food-cue (Volkow et al., 2011). It has been reported that the body weight increases with increasing response of the putamen to food-cue (Cerolini et al., 2017). The putamen activity negatively correlates with weight loss (Berthoud, 2011). Putamen activity decreases in obese patients at one month after sleeve gastrectomy, positively correlating with the BMI reduction (Gu et al., 2020). These reports are consistent with our finding that the putamen activity decreased at endpoint of IER, contributing to weight loss in obese participants.

At endpoint of phase III, we observed significant reductions in the activity of the DLPFC and ACC in the cognitive control network. DLPFC and ACC are involved in inhibition of food intake (Lavagnino et al., 2016), belonging to the “cool- system” that is related to the self-control ability (Metcalfe and Mischel, 1999). DLPFC impairment may increase BMI in healthy adults (Raji et al., 2010). ACC shrinks in obese patients, and the volume of ACC negatively correlates with the ability of subjects to control food intake (Brooks et al., 2013). Structural connection between DLPFC and ACC negatively correlates with the desire for high-calorie food (Hu et al., 2021). In our study, the activity of DLPFC and ACC decreased at the end of lowly-controlled phase III, suggesting that for weight maintenance, it is important to enforce the activity of and the connection between DLPFC and ACC. Long-term follow-up studies have shown significant changes in reward network during weight loss (Murdaugh et al., 2012; Li et al., 2018). However, we did not observe significant alterations in the activity of reward network during the short-term IER intervention, suggesting that the reward network might be involved in a long-term dietary intervention.

As a vital component of the BGM axis, gut microbiota exhibited dynamic alterations in the abundance and diversity during IER in this study. At midpoint of phase II, the abundance of obesity-related probiotics including *F. prausnitzii, P. distasonis, and B. uniformis* significantly increased compared with that at baseline, whereas the abundance of pathogenic *E. coli* significantly decreased. Consistent with our findings, studies have indicated that *E. coli* abundance is related to the obesity pathogenesis and dietary behavior (Nilsson et al., 2001; André et al., 2014; Lima and Fonteles, 2014). Nutritional factors can regulate *E. coli* proliferation, affecting the release of glucagon-like peptide-1 that induces intestinal satiety and dietary termination signals (Peek et al., 2020; Sun et al., 2020). On the contrary, *F. prausnitzii*, *P. distasonis*, and *B. uniformis* can reduce obesity and alleviate obesity-related metabolic and immune disorders (Gauffin Cano et al., 2012; Wang et al., 2019; Schroder et al., 2020). Thus, our findings suggest that the alteration in gut microbiota in response to IER promotes weight loss. Interestingly, the reduction in *E.coli* abundance sustained to the endpoint of phase III, whereas the abundance of *F. prausnitzii*, *P. distasonis*, and *B. uniformis* peaked at midpoint of phase II and declined to near baseline levels at endpoint of phase III. These results demonstrate that gut microbiota of obese individuals rapidly responds to IER at the early stage, consistent with previous reports (Wu et al., 2011; Kusumoto et al., 2017; Asnicar et al., 2021). The rise-and-fall pattern of obesity-related probiotics suggests that gut microbiota is a highly plastic system as indicated by other studies. For example, the Bacteroidetes/Firmicutes ratio is altered within a week by dietary change but remain comparable to baseline level at 2 weeks after dietary intervention (Grembi et al., 2020). Long-term dietary intervention exerts a sustained impact on gut microbiota whereas short-term dietary change only causes rapid and temporary changes in gut microbiota (Wu et al., 2011; Grembi et al., 2020). Thus, for weight loss maintenance, a highly-controlled long-term IER intervention is recommended.

Despite the rigid connection between gut microbiota and the brain in early life, the BGM axis exhibits plasticity in adults, playing a prominent role in regulating food intake and keeping body weight stable (Junges et al., 2018). The chemical signals produced by gut microbiota determine eating behavior through interactions with the brain. Considering the important role of the BGM axis in obesity and weight loss, we explored the association between brain networks and gut microbiota during IER. We observed significant correlations between certain brain regions and differentially abundant gut bacteria across different timepoints of IER. For instance, the *E coli* abundance was negatively associated with the left orbital inferior frontal gyrus in the inhibitory control circuit at base line and the right putamen in the learning and memory circuit at endpoint of phase III. *E. coli* can regulate the anorexia and appetite pathways by penetrating the blood-brain barrier to affect brain function and modulating the expression of appetite-regulating neuropeptides.

## Conclusion

In conclusion, we described a short-term IER-induced dynamic changes in the BGM axis in obese individuals who successfully lost weight after the intervention. We identified the brain regions and differentially abundant gut bacterial species responding to IER at different IER timepoints and revealed a dynamic correlation between the brain and gut microbiota.

### Limitations of the study

The last measurements were taken at 30 days after the IER. Further follow-up study is needed for revealing the BGM axis dynamical alteration which affect the long-term weight loss. In this study, it mainly aims at the specific BGM axis changes during the weight loss under the IER. The results provided primary insights for potential BGM axis changed during weight loss, and did not establish causation. The animal model is need to validate the BGM changes for more understanding about how these certain brain area activity work with specific gut flora in the weight loss.

## Data availability statement

The raw sequence data reported in this paper have been deposited in the Genome Sequence Archive (Genomics, Proteomics & Bioinformatics 2021) in National Genomics Data Center (Nucleic Acids Res 2022), China National Center for Bioinformation / Beijing Institute of Genomics, Chinese Academy of Sciences (GSA: CRA013299) that are publicly accessible at https://ngdc.cncb.ac.cn/gsa.

## Ethics statement

The studies involving humans were approved by the Ethics Committee of Henan Provincial People’s Hospital, People’s Hospital of Zhengzhou University, and Zhengzhou People’s Hospital (Henan, China). The studies were conducted in accordance with the local legislation and institutional requirements. The participants provided their written informed consent to participate in this study.

## Author contributions

JZ: Conceptualization, Data curation, Formal Analysis, Writing – original draft. XLW: Investigation, Methodology, Writing – original draft. TX: Formal Analysis, Methodology, Writing – review & editing. FL: Formal Analysis, Methodology, Software, Writing – review & editing. HG: Conceptualization, Investigation, Methodology, Supervision, Writing – review & editing. LT: Formal Analysis, Supervision, Validation, Writing – review & editing. BY: Formal Analysis, Project administration, Validation, Writing – review & editing. ZLL: Formal Analysis, Methodology, Writing – review & editing. CZ: Project administration, Resources, Writing – review & editing. LYW: Formal Analysis, Investigation, Methodology, Writing – review & editing. LO: Software, Writing – review & editing. ZXL: Data curation, Validation, Writing – review & editing. WW: Investigation, Resources, Writing – review & editing. TY: Investigation, Methodology, Writing – review & editing. FYL: Supervision, Writing – review & editing. HM: Supervision, Writing – review & editing. XZ: Data curation, Investigation, Writing – review & editing. NM: Investigation, Writing – review & editing. ZY: Investigation, Methodology, Writing – review & editing. CL: Supervision, Validation, Writing – review & editing. QW: Supervision, Validation, Writing – review & editing. HL: Conceptualization, Investigation, Resources, Supervision, Writing – review & editing. LMW: Conceptualization, Formal Analysis, Supervision, Writing – review & editing. XNW: Conceptualization, Supervision, Validation, Writing – review & editing. YL: Conceptualization, Funding acquisition, Supervision, Writing – review & editing. QZ: Conceptualization, Funding acquisition, Supervision, Writing – review & editing.
