# Supplementary material for: Dynamical alterations of brain function and gut microbiome in weight loss
Source: Front Cell Infect Microbiol. 2023 Dec 20;13:1269548. doi: 10.3389/fcimb.2023.1269548 (PMC10761423; doi:10.3389/fcimb.2023.1269548)
Supplement: Supplementary file 1 [file DataSheet_1.docx]

**
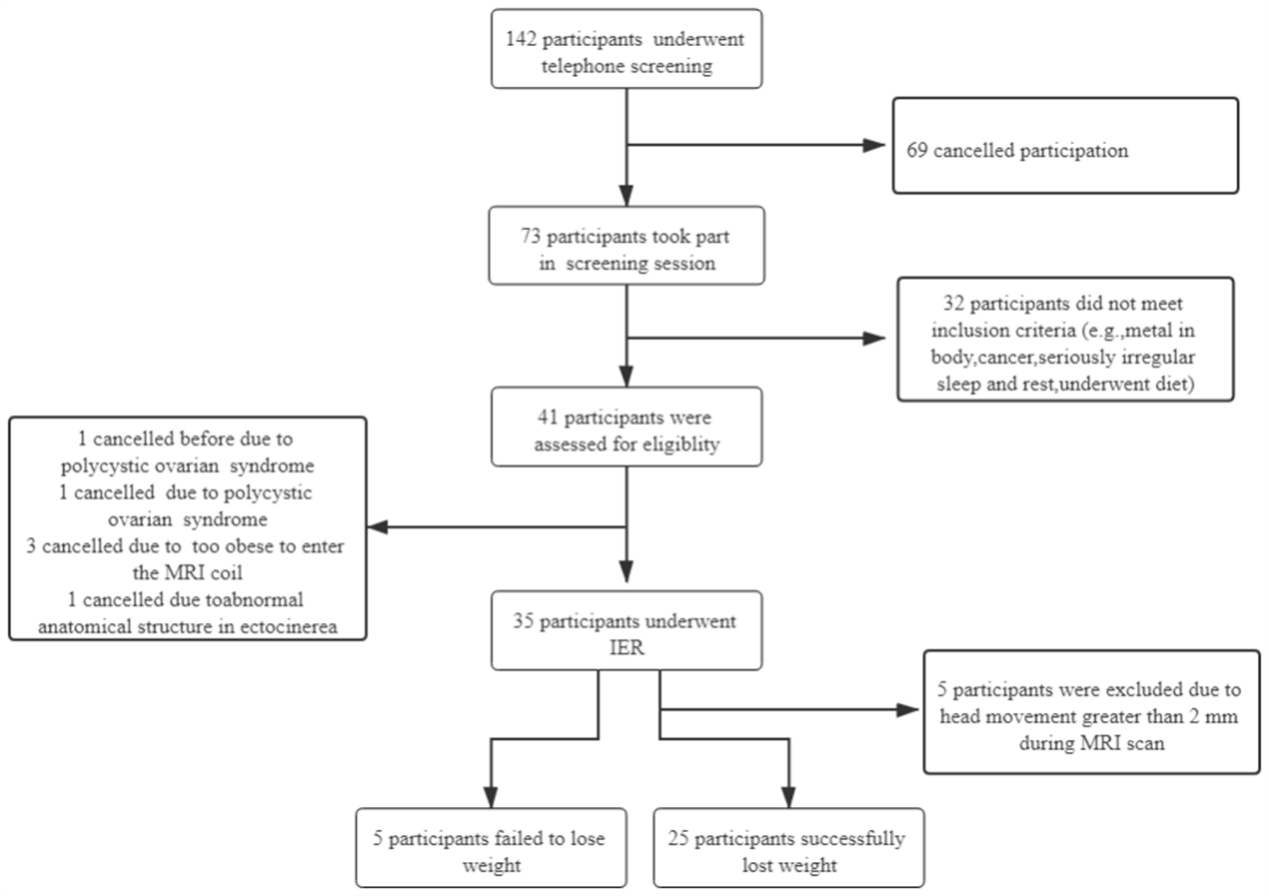
**

**Figure S1. A schematic overview of recruitment of participants.**

**
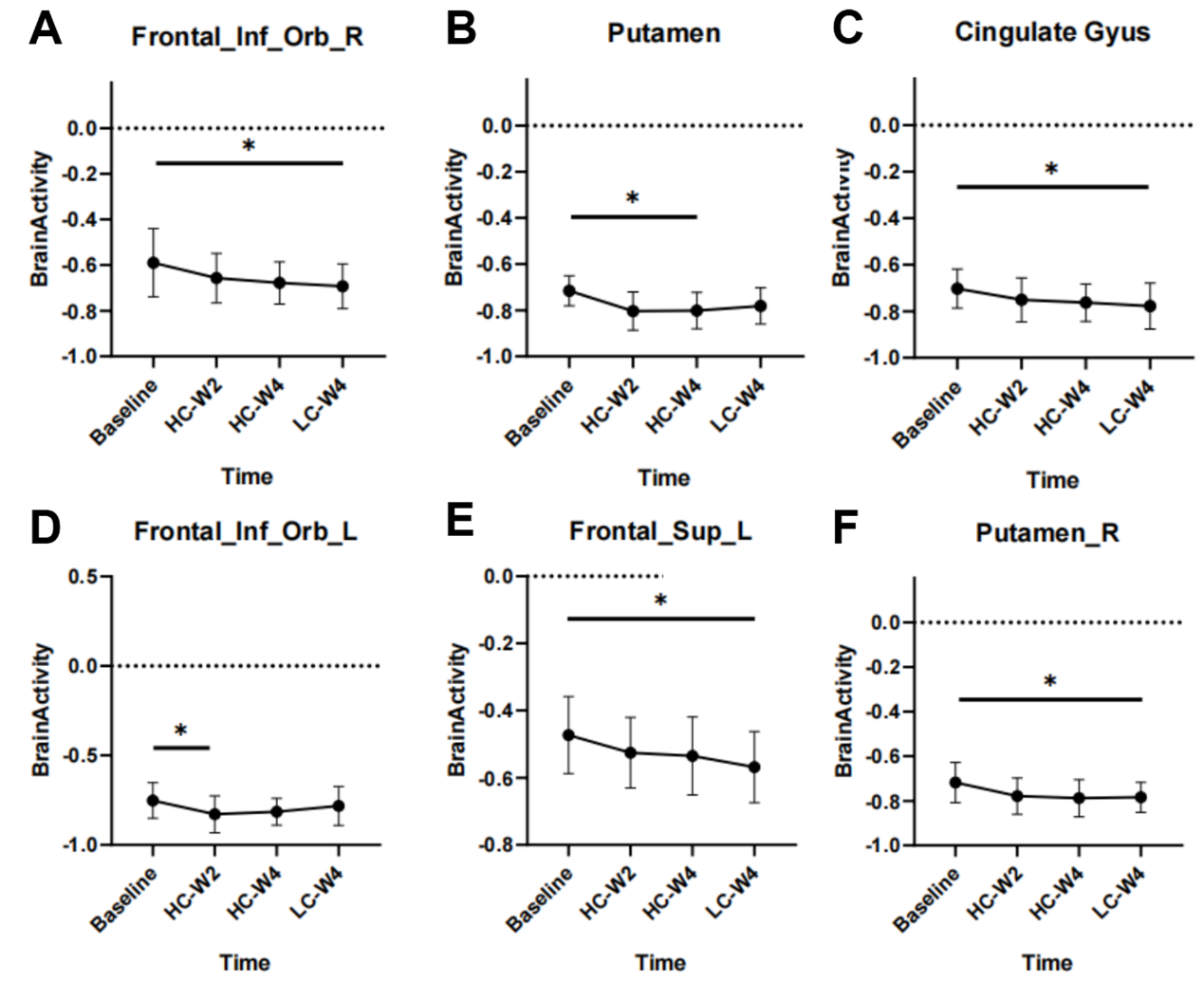
**

**Figure S2.** **Dynamic changes in brain activity during IER.** The activity of right inferior frontal orbital gyrus (A), putamen (B), anterior cingulate cortex (C), left inferior frontal orbital gyrus (D), left dorsolateral prefrontal cortex (E), and right putamen were assessed by fMRI at different IER timepoints. **P* < 0.05.

**Table S1.** The sequence of four brain network activation changes over the process of IER.

| **Time points** | **Brain region** | **MNI Coordinates** | | | **T value** |
| --- | --- | --- | --- | --- | --- |
|  |  | **X** | **Y** | **Z** |  |
| HC-W2--baseline | Frontal_Inf_Orb_L | -15 | 15 | -24 | -4.246 |
| HC-W4--baseline | Putamen | 30 | -21 | 0 | -4.714 |
| LC-W4--baseline | Putamen_R | 27 | 18 | 9 | -4.474 |
|  | Frontal_Inf_Orb_R | 27 | 30 | -9 | -5.112 |
|  | Cingulate Gyrus | 15 | 15 | 36 | -6.425 |
|  | Frontal_Sup_L | -12 | 1 | 45 | -5.267 |

ReHo analysis was corrected by GRF (P < 0.005).
